# Speed but Not Smoothness of Gait Reacts to Rehabilitation in Multiple Sclerosis

**DOI:** 10.1155/2021/5589562

**Published:** 2021-06-03

**Authors:** Philipp Gulde, Joachim Hermsdörfer, Peter Rieckmann

**Affiliations:** ^1^Centre for Clinical Neuroplasticity Medical Park Loipl, Bischofswiesen, Germany; ^2^Technical University of Munich, Munich, Germany

## Abstract

**Introduction:**

Improved gait is one of the leading therapy goals in multiple sclerosis. A plethora of clinical timed trials and state-of-the-art technology-based approaches are available to assess gait performance.

**Objectives:**

To examine what aspects of gait react to inpatient rehabilitation in MS and which parameters should be best assessed.

**Design:**

In this longitudinal study, we examined the performance of 76 patients with MS to shed further light on factors influencing gait, associations between tests, and the reaction to inpatient rehabilitation during an average time span of 16 d. *Setting*. Private specialist clinic for inpatient neurorehabilitation. *Main Outcome Measures*. Clinical walk tests (timed 25-foot walk test at normal pace, maximum pace over 10 m or 6 min) and IMU-based measures of movement smoothness.

**Results:**

All gait parameters were strongly intercorrelated (all *p* < 0.05), and a model multiple linear regression for the 6MWT revealed short distance velocity (10 m) and movement smoothness as predictors in a strong model (*R*^2^_adjusted_ 0.75, *p* < 0.01). A second model with natural pace on short distance and movement smoothness was almost equally strong (*R*^2^_adjusted_ 0.71, *p* < 0.01). Patients improved their walking speed (*p* < 0.01), but not smoothness (*p* = 0.08–0.12), over the course of rehabilitation.

**Conclusions:**

Since we were not able to observe improvements in smoothness of gait, we conclude that rehabilitation programs should be adapted to the patient's physiological capacities in order to allow for such improvements in smoothness of gait. Externally valid gait capacity (6MWT) could be predicted by a single walk for 10 s at natural pace.

## 1. Introduction

Multiple sclerosis (MS) is a centrally demyelinating autoimmune disease that affects approx. 1-2 in 1000 in central Europe [1, 2]. The clinical severity of MS is commonly rated by the expanded disability status scale [3, 4], a coarse estimate with emphasis on gait. Patients, especially close to their first disease onset, usually report the loss of gait to be one of their most troubling fears [5]. Therefore, interventions dominantly aim at the walking ability [6–10]. In order to estimate the effectiveness of such interventions, a variety of classic clinical timed trials [9, 11–15] as well as a range of innovative, state-of-the-art kinematic and dynamometric assessments are being used in (neurological) rehabilitation and aging [16–24].

When assessing walking capacity, two questions arise. What is being assessed? And what is improving due to (multidisciplinary) therapy? In multiple sclerosis, there is very little data on the effects of inpatient rehabilitation on functional capacity in gait, neither on speed, coverable distance, nor on profiting dimensions. However, there are data on exercise interventions on gait. One commonly used approach is resistance training, showing effects of 10% to 25% of improvements on short distance tests (10 m walk test (10MWT) and timed stand up and go) over the course of several weeks to months (56-84 days) [25–27]. A meta-analysis by Pearson et al. [9] showed mean effects of -1.76 s (19% improvement) in the 10MWT and +36.5 m in the 6 min walk test (6MWT) with a subgroup analysis for the 10MWT that revealed no effects of intervention type (resistance training, aerobic training, yoga, or mixed). The impact of the EDSS as clinical severity of MS remained unclear.

In this study, we assessed MS patients with a variety of classic clinical gait tests and an inertial measurement unit- (IMU-) based approach in a longitudinal manner. We hypothesized that gait performance was a combination of physical capacity and adapted skills (to such a physical capacity). Further, we anticipated improvements in both dimensions due to multidisciplinary inpatient rehabilitation. A prior study assessing only smoothness of gait revealed a very weak effect of rehabilitation in MS patients [24]. Here, we extend those findings by a more comprehensive set of outcome parameters. This study was part of the “Implementation of a Neuro Assessment Lab” project.

### 1.1. Objectives and Hypotheses

We wanted to examine the effects of inpatient rehabilitation on gait capacity in MS and explore which parameters are most appropriate to assess in order to display gait capacity and its changes due to rehabilitation. We hypothesized that two observable dimensions of gait (physical capacity and adapted skills) would improve over the course of inpatient rehabilitation.

## 2. Methods

### 2.1. Sample

A total of 76 patients (34% male, 66% female), with a mean age of 50.8 yrs ± 10.3 yrs (26-72 yrs), was included in this study. Time since first diagnosis was 13.3 yrs ± 8.7 yrs (0-40 yrs). The mean EDSS of patients was 3.9 ± 1.7 (1.0-7.0, median: 4.0). 53% of the cases were categorized as relapsing remitting, 17% as primary progressive, 26% as secondary progressive, and 4% as a first onset and/or nonclassified MS. Patients were recruited at the Centre for Clinical Neuroplasticity, Medical Park Loipl (Medical Park Group) in Germany.

Inclusion criteria were patient's consent, 18 yrs of age, and a diagnosed MS. Exclusion criteria were the inability to walk and the inability to give consent (e.g., severe cognitive impairment), bone fractures and injuries, acute infections (e.g., a cold), strong adverse effects of medication, and the presence of other neurological diseases (e.g., stroke).

All patients gave written informed consent. Ethical approval was given by the ethics committee of the Medical Faculty of Technical University of Munich.

### 2.2. Clinical Gait Tests

Patients performed the timed 25-foot walk at a “natural pace,” which was assessed by the main author of the study. A “natural pace” was chosen to be able to examine an estimated ecological valid behavior in comparison to the maximum physical capacity of the patient (assessed by the 10MWT). Further, the 10MWT and the 6MWT were both assessed by therapists associated with the rehabilitation facility. In these tests, patients were instructed to walk “as fast as possible.” For all tests, the velocity was derived. Additionally, the therapist counted the number of steps during the 10MWT, so step frequency and step length could be estimated. The 10MWT and 6MWT are both clinical standards and offer the opportunity to additionally evaluate the practicability and reliability of these tests. The therapists were blinded to the study outcomes, but were aware that changes in walking speed are commonly used as a criterion of quality of rehabilitation.

### 2.3. Digital Gait Test

A Microsoft Lumia 550 smartphone was attached to the patients' sternum with a chest belt. Participants were ask to walk at a “natural pace,” and the scalar of the three acceleration axes of the smartphone's IMU was recorded over a time span of 10 s at approx. 20 Hz. Two parameters of movement smoothness were derived [24, 28].

**Figure 1:**
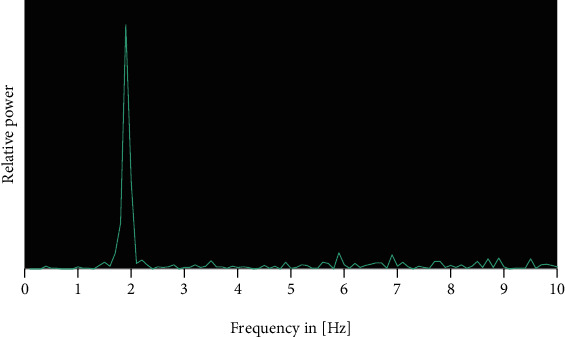
Frequency spectrum of the acceleration at the sternum during a 10 s walk at natural pace. The step frequency was 1.8 Hz. Complexity was 62%.

The complexity of the signal by using the ratio of arc length and integral of the frequency spectrum ranges from 0 to 10 Hz (steps of 0.1 Hz) in comparison to the ratio of a frequency spectrum using only one frequency, resulting in a percentage between 0 and 100%, with 100% being the highest possible smoothness (derived from the spectral arc length smoothness measure of [29]) (Figure 1).

**Figure 2:**
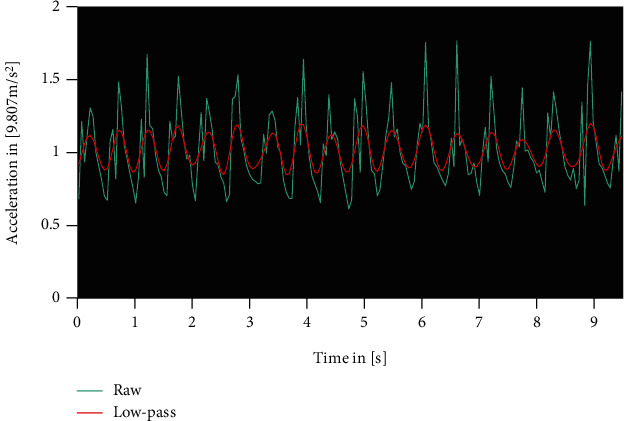
Raw acceleration signal in turquoise and 3 Hz low-pass filtered signal in red. The noise (*R*^2^) was 65%.

The second parameter estimated the signal-to-noise ratio by the coefficient of determination between the raw signal and a 3 Hz low-pass filtered signal, resulting in smoothness levels between 0 and 100%, with 100% being the highest possible smoothness (Figure 2). For the frequency analysis and low-pass filtering, discrete Fourier transformations were applied forwards and backwards.

Both measures have already been successfully applied in an MS sample revealing strong correlations with the EDSS (*r*_complexity_ = −0.87, *r*_noise_ = −0.82) [24].

As a third outcome, the step frequency was guessed as the frequency with the highest power. It was not possible to reliably estimate the movement velocity by IMUs, so we relied on timed walk tests.

The software was developed using UWP C# (Microsoft Visual Studio 2017, Microsoft Cooperation).

Results of such a gait assessment in a cohort of MS patients, showing strong associations with the EDSS in both smoothness parameters, had already been presented [21, 24].

### 2.4. Physical Capacity and Adapted Skills

By this test battery, we anticipated to collect data on the physical capacity (maximum velocity over 10 m or over 6 min, step length and frequency) and adapted skills (smoothness of gait).

### 2.5. Procedure

Patients were assessed twice, once close to their entry (6.7 d ± 5.4 d) and a second time close to their release. The average time span between measurements was 16.1 d ± 5.9 d (4-28 d). During their inpatient rehabilitation, patients received no interventions additional to their schedule, which was a multidisciplinary treatment following international guidelines.

In the first session, all gait tests were performed, while during the second session, only the 10MWT, the 6MWT, and the digital assessment were performed to examine changes in short- and long-distance speed and smoothness of gait due to rehabilitation.

### 2.6. Parameter Abbreviations

EDSS: an estimate of the clinical severity of MS by a physician on a rating scale from 0.0 to 10.0, which is based on the severity and combination of neurological symptoms and gait capacity.

10MWT: the maximum velocity over a 10 m distance (m/s).

10MWT Freq: the estimated step frequency during the 10 m walk test (Hz).

10MWT Step: the estimated step length during the 10 m walk test (m).

6MWT: the velocity that could be maximally kept over 6 min (m/s).

T25FW: the velocity over a 7.62 m distance at a natural pace (m/s).

Freq: the digitally assessed step frequency (Hz).

Step: estimated step length at a natural pace by 7.62 m/(T25FW_time_ × Freq) (m).

Complexity: complexity of acceleration signal when walking at a “natural pace” (%).

Noise: 3 Hz signal-to-noise relation when walking at a “natural pace” (%).

### 2.7. Statistical Analysis

Required sample sizes were calculated by G∗Power 3.1 [30]. We anticipated small effects of approx. 0.33 based on gait improvements of stroke survivors in the same rehabilitation facility [31]. The resulting required sample size was 75 with *α* = 0.05 and a power of 0.80.

Paired *t*-tests were computed for each parameter between the first and second assessments. Effect sizes were estimated by Glass' *Δ* (based on entry stats). Associations were estimated by Pearson correlations. Correlations with EDSS and age used an EDSS-adjusted age and an age-adjusted EDSS due to their correlation (higher EDSS grades in higher age). A model of multiple linear regression was built to predict the 6MWT performance by all other gait parameters. A principal component analysis (PCA) was computed to examine for gait parameter associations. The critical *α* for statistical significance was set to 0.05. No *α*-corrections for correlated tests were applied due to the risk of *β*-inflations. The critical variance inflation (VIF) was set to <5.0. Statistical analyses were performed with RStudio (RStudio Inc.).

## 3. Results

### 3.1. Associations with Age and EDSS

Only 10MWT Step revealed a significant association with the EDSS-adjusted age of patients (*r* = −0.25, *p* < 0.05). All gait parameters showed significant correlations with the age-adjusted EDSS, whereas 10MWT Step, Step, and Freq had the lowest coefficients and all other parameters were within a range of *r* = 0.55–0.66 (Table 1).

### 3.2. Parameter Associations

**Figure 3:**
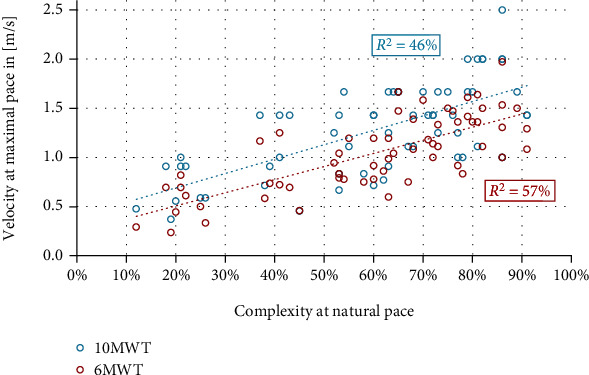
Scatter plot of complexity at natural pace and 10MWT as well as 6MWT velocity at maximal pace.

All gait parameters were significantly intercorrelated (all *p* values < 0.05) (Table 2, Figure 3). The strongest associations were observed between clinical gait tests. A principal component analysis revealed only one component with an eigenvalue of 6.55 and 73% explained variance (all other components had eigenvalues < 1.00, Kaiser-Meyer-Olkin criterion was 0.65, minimum measure of sample adequacy was 0.50, Bartlett test for sphericity *p* < 0.01, anti-image was 1.00 in all cases, and component communalities were all <0.14; *n* = 51).

### 3.3. 6MWT Model

**Figure 4:**
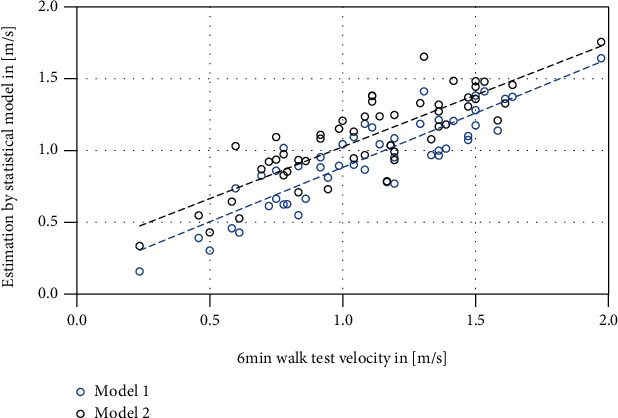
Scatter plot of the models of multiple linear regression to predict the 6MWT velocity. Model 1 (blue), using 10MWT and complexity, had an *R*^2^_adjusted_ of 0.75. Model 2 (black), using T25FW and complexity, had an *R*^2^_adjusted_ of 0.70. The factors for model 2 can be assessed by a single walk of 10 s.

A model of multiple linear regression to predict the 6MWT performance showed two significant factors: 10MWT and complexity, with *β*-weights of 0.58 and 0.37. The model had *R*^2^_adjusted_ of 0.75 (*p* < 0.01, Table 3, Figure 4), with 6MWT = −0.016 + 0.461∗10MWT + 0.751∗complexity. An alternative model with *R*^2^_adjusted_ of 0.71 (*p* < 0.01) used the velocity (T25FW) in normal pace and complexity (Table 4) with the resulting formula 6MWT = −0.037 + 0.691∗T25FW + 0.575∗complexity.

### 3.4. Pre-Postcomparison

A comparison of performance of all parameters but T25FW and Step showed significant changes over 16.1 d ± 5.9 d between assessments. All parameters that were assessed by the therapist associated with the rehabilitation facility and the digitally guessed step frequency showed improvements with weak effect sizes (Table 5).

### 3.5. Pre-Postcorrelations

Correlations between changes in gait parameters showed strong coefficients between 6MWT and 10MWT markers and between 10MWT Freq and 10MWT. Correlations with digitally assessed parameters of gait were nonsignificant to weak (Table 6).

## 4. Discussion

In this study, 76 patients with multiple sclerosis were examined in a variety of clinical timed trials and digitally assessed gait tests. Performance was reassessed after an average of 16 d in a facility for neurological rehabilitation.

### 4.1. Associations with Age and EDSS

All parameters but 10MWT Step (step length derived from the 10MWT) were independent from the EDSS-adjusted age of patients and revealed moderate-to-strong associations with the age-adjusted EDSS, as already published, e.g., by Behrens et al. [16]. Digitally assessed and clinical tests showed to be equally strong in their coefficients. The missing association with the EDSS-adjusted age could be an indicator of an altered ageing process due to the disease or an altered disease progression due to the age [32]. The associations of complexity and noise with the EDSS_age adjusted_ in our sample were lower than in a previous study [24]. Varying coefficients of correlation can derive from differences in the distribution of EDSS levels [33], the scale characteristics (partially superimposition of ordinal scales), the resulting statistical behavior with reduced reliability [34], the measurement error of the test [33], and the interaction between disease progression and subscores of the EDSS (e.g., pyramidal or cerebellar as predictors of quicker EDSS progression [35]).

### 4.2. Parameter Associations

The strong intercorrelations and the one-component result of a PCA indicate that all parameters were basically assessing the same nonspecific (a single factor with a high eigenvalue) dimension of “walking,” since the parameter loadings on this component were all between -0.30 and -0.37.

### 4.3. 6MWT Model

When predicting the 6MWT performance—as a parameter that could be viewed as externally valid [15]—two factors showed significant impact, the 10MWT velocity and complexity, as a measure of movement smoothness. Although the model indicated some collinearity, the implications are clear. A gait pattern that can be sustained over 6MWT comprises a certain physical or “sprint capacity” (maximum velocity of 10MWT) and economic, smooth kinematics of the body mass, which fit our hypothesis and the results of, e.g., Kieseier & Pozzilli [12], although the PCA rather indicated that this model could be far too simple. In future studies, more biomechanical markers like lower-limb force production should be added in order to get a better picture of impaired gait. However, the option to assess the 6MWT performance by two quick assessments in a single “run” over approx. 10 s (the second model) could be seen as beneficial for patient and therapist. Such an alternative could also limit a bias towards improvement in assessments by the treating therapist; we observed an improvement in the 6MWT in 81% of therapist reports, while the rate was 60% in our statistical model.

### 4.4. Pre-Postcomparison and Correlations

The pre-postcomparison of parameters and correlations between improvements in gait metrics revealed a potentially troubling picture of rehabilitation. If increased velocities, step lengths, and step frequencies could be observed without an increment of movement smoothness, one could argue that patients habituate and learn to walk faster on the basis of an increased risk-taking behavior. Since the step frequency changes during a “natural pace” were in range, and moderately-correlated, with the improvements in therapist-derived frequencies, as well as first assessment step lengths were well-associated, the data appears reliable enough (although the 6MWT velocity was faster than the 10MWT velocity in 12%_first assessment_ and 17%_second assessment_ of the cases) to draw the conclusion that patients were adapting a gait behavior that was ultimately not well fit to their physical capacities. A study on fall risk in MS, too, revealed that variability in gait was higher in fallers than nonfallers and correlations with, e.g., T25FW was only moderate, while stronger with more complex tasks like the timed stand up and go task [36]. Further, Sosnoff et al. [37] reported walking coordination and endurance, but not speed, to be associated with MS patients that fell within the last 12 months. In this sense, we observed a change in tests assessing physical capacity, but not in parameters assessing adapted skills. Since those parameters were strongly associated in the first assessment, a unidimensional change due to rehabilitation seems questionable and rather suggests the abovementioned increase in risk-taking behavior during walk or walk tests, respectively. Interestingly, a previous study [24] showed a very weak, significant effect of rehabilitation on noise (*p* < 0.01, *d* = 0.18), but not on complexity (*p* = 0.10). Potential explanations could be either the assumption that rehabilitation can be almost unpredictable [24] due to multiple factors potentially impacting the effects (therapist, patient, environment and facility equipment, content of schedule, treating physicians, seasonal effects, and so on), a large measurement error in the assessment of noise (but potentially not complexity), or the slightly different sample characteristic including higher EDSS grades in Gulde et al. [24] (a mean of 4.4 and a range of 1.0 to 8.5). As Baert et al. [33] have shown, changes in gait capacity can be dependent on disability level and can be further very heterogenic, so large samples seem warranted.

### 4.5. Limitations

As discussed, there was a questionable objectivity in data that were assessed by associated therapists, including a bias towards improvement of their patients (assessment and treatment by the same person). Although estimating 71% of variance of the 6MWT within 10 s is high, it should be kept in mind that the residual standard error of 0.19 m/s equals approx. 70 m (CI_95_ = [−155 m, 137 m], with the residual being positively correlated with the 6MWT performance (*r* = 0.53, *p* < 0.01)), so the detection of change (0.18 m/s) could be still quite limited. As it has been shown that other gait measures show better sensitivity to changes in performance [33], the effects of rehabilitation in this study could have been mis- or even underestimated. However, the high proportion of explained variance of the 6MWT could serve to a certain degree as a measure of reliability of our data (although assessments were not blinded), but the overall limited external validity of lab measurements of gait velocity should always be kept in mind [38]. Further, we did not test the model by a test data set, so its generalizability is so far not supported by evidence. As an additional limitation of the study, it has to be mentioned that the between-session interval was relatively short with averagely 16 d. Although the variance in time to the first session and interval between sessions holds some interesting information concerning temporal dose-response relationships, studies with expanded protocols are warranted in order to be able to more sensitively investigate rehabilitation of gait.

## 5. Conclusion

From the present results, we conclude that gait tests can be validly applied in MS patients, most tests are assessing similar, but different, aspects of the walking ability (see PCA), and inpatient rehabilitation might need to further adapt its methodology and pattern of movement therapy to the physiological capacities of patients in order to not only improve gait velocity and distance (by velocity) but also allow economic and smooth kinematics of the body mass (rehabilitation should increase physical capacity and (further) adapt patients' skills). We advise to use a mixed assessment strategy, comprising movement smoothness and short-distance velocity (10MWT velocity and IMU-derived movement smoothness or velocity and movement smoothness at natural pace) that can be assessed in less than a minute or, in case of the second, statistical slightly weaker model within a single walk of 10 s at natural pace. By introducing movement smoothness as a measurable therapy goal, one could automatically shift the attention from sheer velocity to a gait pattern that is adapted to the physical capacities and the specific strengthening of physiological supportive dimensions like increased strength, reduced spasticity, or well-developed proprioception and sensorimotor integration. Finally, it has to be kept in mind that this study suffers from limitations of objectivity, so results should be taken with a certain caution.

## Figures and Tables

**Table 1:** Coefficients of correlation between gait parameters and the EDSS-adjusted age as well as age-adjusted EDSS of patients. ^∗^*p* < 0.05.

	10MWT	10MWT Freq	10MWT Step	6MWT	T25FW	Freq	Step	Complexity	Noise
Age_EDSS adjusted_	-0.14	0.05	-0.25^∗^	-0.10	-0.22	-0.07	-0.23	-0.07	-0.03
EDSS_age adjusted_	-0.60^∗^	-0.62^∗^	-0.48^∗^	-0.66^∗^	-0.63^∗^	-0.46^∗^	-0.48^∗^	-0.63^∗^	-0.55^∗^

**Table 2:** Coefficients of correlation between all gait parameters. All *p* values < 0.05.

	10MWT	10MWT Freq	10MWT Step	6MWT	T25FW	Freq	Step	Complexity
10MWT Freq	0.88							
10MWT Step	0.91	0.63						
6MWT	0.87	0.76	0.82					
T25FW	0.89	0.80	0.81	0.86				
Freq	0.62	0.71	0.50	0.66	0.70			
Step	0.75	0.55	0.74	0.65	0.89	0.32		
Complexity	0.68	0.64	0.62	0.75	0.80	0.72	0.56	
Noise	0.63	0.48	0.64	0.64	0.70	0.52	0.56	0.83

**Table 3:** Prediction of 6MWT by 10MWT and complexity. The model had an *R*^2^_adjusted_ of 0.75 with *p* < 0.01.

	10MWT	Complexity
*β*-Weight	0.58	0.37
VIF	1.76	1.76
*p*	<0.01	<0.01

**Table 4:** Prediction of 6MWT by T25FW and complexity. The model had an *R*^2^_adjusted_ of 0.71 with *p* < 0.01.

	T25FW	Complexity
*β*-Weight	0.62	0.28
VIF	2.38	2.38
*p*	<0.01	0.02

**Table 5:** Comparison of first and second assessment performances in all gait parameters but T25FW and Step.

	Pre	Post	Change
10MWT *n* = 64 | 64	1.24 m/s ± 0.49 m/s	1.43 m/s ± 0.55 m/s	*p* < 0.01 Glass' Δ = 0.39
10MWT Freq *n* = 64 | 64	1.89 Hz ± 0.43 Hz	1.98 Hz ± 0.47 Hz	*p* = 0.038 Glass' Δ = 0.22
10MWT Step *n* = 64 | 64	0.64 m ± 0.15 m	0.71 m ± 0.17 m	*p* < 0.01 Glass' Δ = 0.46
6MWT *n* = 64 | 64	1.01 m/s ± 0.41 m/s	1.18 m/s ± 0.46 m/s	*p* < 0.01 Glass' Δ = 0.43
Freq *n* = 66 | 57	1.64 Hz ± 0.33 Hz	1.73 Hz ± 0.35 Hz	*p* = 0.025 Glass' Δ = 0.28
Comp *n* = 73 | 61	60% ± 22%	62% ± 22%	*p* = 0.118
Noise *n* = 69 | 59	68% ± 16%	70% ± 14%	*p* = 0.084
T25FW *n* = 69 | 0	1.03 m/s ± 0.36 m/s	n.a.	
Step *n* = 61 | 0	0.65 m ± 0.14 m	n.a.	

**Table 6:** Coefficients of correlation between improvements in gait parameters. ^∗^*p* < 0.05.

	Complexity_Improvement_	Noise_Improvement_	10MWT Freq_Improvement_	10MWT_Improvement_	6MWT_Improvement_
Freq_Improvement_	0.52^∗^	0.36^∗^	0.42^∗^	0.30^∗^	-0.02
Complexity_Improvement_		0.19	0.14	0.09	0.02
Noise_Improvement_			0.19	0.27^∗^	0.03
10MWT Freq_Improvement_				0.93^∗^	0.64^∗^
10MWT_Improvement_					0.62^∗^

## Data Availability

Data is available on request to the corresponding author.
